# Differential Effects of Pregnancy-Specific Alcohol Policies on Drinking Among Pregnant Women by Race/Ethnicity

**DOI:** 10.1089/heq.2018.0059

**Published:** 2018-12-13

**Authors:** Sarah C.M. Roberts, Amy A. Mericle, Meenakshi S. Subbaraman, Sue Thomas, Ryan D. Treffers, Kevin L. Delucchi, William C. Kerr

**Affiliations:** ^1^ Advancing New Standards in Reproductive Health (ANSIRH), Department of Obstetrics, Gynecology, and Reproductive Sciences, University of California, San Francisco, Oakland, California.; ^2^ Alcohol Research Group, Public Health Institute, Emeryville, California.; ^3^ Pacific Institute for Research and Evaluation (PIRE), Santa Cruz, California.; ^4^ Department of Psychiatry, University of California, San Francisco, San Francisco, California.

**Keywords:** alcohol, alcohol policy, binge drinking, pregnancy

## Abstract

**Purpose:** Alcohol use during pregnancy is a significant public health concern. Nearly all U.S. states have enacted policies targeting alcohol use during pregnancy, but there has been little research examining their impact, particularly across racial/ethnic groups.

**Methods:** Using data from the Behavioral Risk Factor Surveillance System and about eight state-level, pregnancy-specific alcohol policies from 1985 to 2016, the aim of this study was to examine the differential effects of these policies on drinking among pregnant women by race/ethnicity.

**Results:** We found evidence of differential effects for priority treatment, prohibitions on criminal prosecution, and civil commitment policies. In relation to priority treatment policies, effects benefited versus harmed different racial/ethnic groups depending on whether the priority treatment policies were for pregnant women only or if they gave priority to both pregnant women and pregnant women with children.

**Conclusions:** Findings from this study suggest that benefits and harms from these policies do not appear to be equitably distributed across different racial/ethnic groups. Research considering the impact of alcohol/pregnancy policies should consider differential effects by race/ethnicity.

## Introduction

Alcohol is a well-established teratogen, causing multiple harms, including fetal alcohol spectrum disorders.^1^ There is no known safe level of alcohol use during pregnancy; women who drink heavily and in binge patterns are at higher risk of adverse effects.^2,3^ Use during pregnancy is common; about 15% of U.S. pregnant women report any alcohol use, and almost 3% report binge drinking.^4^ Rates of alcohol use during pregnancy declined in the late 1980s, but have remained relatively stable since 1991.^5–9^ National data mask variation in prevalence of alcohol use during pregnancy across states and in directions of trends across states.^10^ Such variation suggests that state-level policies may play a role.

In 1974, Massachusetts became the first U.S. state to enact a policy targeting alcohol use during pregnancy. By 1990, 20 states had at least one alcohol/pregnancy policy; by 2013, the number of states grew to 43.^11^ These policies include: mandatory warning signs (MWS) in establishments that sell or serve alcohol to warn about impacts of alcohol use during pregnancy; priority treatment for pregnant women (PTP) using or abusing alcohol; prohibitions against criminal prosecution of women who expose fetuses to alcohol (PCP); requirements to report alcohol use or abuse by pregnant women for Data Treatment purposes (RRDTx) or for child protective services (RRCPS); use of indicators of alcohol use during pregnancy as evidence of child abuse or neglect (CACN); and civil commitment (CC) of pregnant women who use or abuse alcohol.^11^

Scholars have characterized alcohol/pregnancy policies as supportive or punitive (Table 1).^12,13^ Supportive policies provide information, early intervention, or services. Punitive policies use coercion to compel behavior change. Both types could reduce drinking, but punitive policies could also deter pregnant women from disclosing use to those who could help them reduce or stop drinking.^14,15^ Although both supportive and punitive alcohol/pregnancy policies have increased over time, state-level policy environments have become increasingly punitive.^11^

**Table 1.** Summary of Pregnancy-Specific Alcohol Policies by Policy Type

Policy	Year first in effect	State(s) where first in effect	No. of states with policy in effect (2016)	Specific states with policy in effect (2016)
Supportive				
MWS	1985	DC	24	AK, AZ, CA, DE, DC, GA, IL, KY, MN, MO, NE, NV, NH, NJ, NM, NY, NC, OR, SD, TN, TX, UT, WA, WV
PTP	1989	CA	11	AK, AZ, AR, CA, CO, GA, KS, KY, OK, UT, WI
PTP+WC	1989	FL, WA	4	DC, IL, MO, WA
RRDTx	1986	KS	27	AK, CA, CO, DE, FL, IL, IN, KS, KY, ME, MI, MN, MO, NV, NJ, NY, ND, OH, OK, OR, PA, SC, SD, TX, WA, WV, WI
PCP	1992	KY, MO, VA	7	CO, KS, KY, LA, MO, NV, VA
Punitive				
CC	1998	SD, WI	5	MN, ND, OK, SD, WI
CACN	1974	MA	21	AL, AZ, CO, GA, FL, IL, IN, KY, LA, ME, MA, NV, ND, OK, RI, SC, SD, TX, UT, VA, WI
RRCPS	1974	MA	21	AK, AZ, AR, CA, DC, FL, IN, KY, LA, ME, MD, MA, MI, MN, OK, PA, RI, SD, UT, VA, WI

MWS, mandatory warning signs; PTP, priority treatment for pregnant women; PTP+WC, priority treatment for pregnant women + women with children; RRDTx, reporting requirements for data and treatment purposes; RRCPS, Reporting requirements for CPS purposes; PCP, prohibitions on criminal prosecution; CACN, child abuse/neglect; CC, civil commitment.

Despite the fact that alcohol/pregnancy policies have been in effect for 40 years, little research examines their impact. Cil found evidence suggesting that MWS may be associated with decreased drinking during pregnancy.^16^ Our own research found that although the intent of alcohol/pregnancy policies may be to reduce drinking during pregnancy, very few policies do, and there is little consistency across drinking outcomes. MWS and CACN policies were associated with decreased drinking during pregnancy, but PTPWC was associated with *increased* drinking.

Prior research has not examined potential differential effects of alcohol/pregnancy policies across race/ethnicity. Previous findings for the whole population could mask differential effects, showing up as no overall effect because effects are in opposite directions or because policies only affect subgroups. Investigating potential differential effects is also important because research has emphasized that population-level interventions that improve health overall do not necessarily reduce (and may increase) disparities across racial/ethnic groups.^17^ This study examines differential effects of alcohol/pregnancy policies on drinking among pregnant women by race/ethnicity.

Our study explores several general hypotheses. Approaches to population health that rely on individual agency and ability to apply knowledge and information to one's own behaviors, such as warning signs, may increase disparities.^18–20^ In addition, being reported to CPS and having a child removed is more common among Black than White and Hispanic women.^21–23^ Fears about CPS may thus differentially decrease disclosure of alcohol use in settings where disclosure may facilitate access to treatment and other services, thereby leading Black and Hispanic women to be less likely to receive help. Thus, we expect that health benefits of alcohol/pregnancy policies (i.e., decreased alcohol consumption during pregnancy) will be greatest among White women and that, to the extent that alcohol/pregnancy policies have negative health effects (i.e., increased alcohol consumption during pregnancy), they will be greatest among Hispanic and Black women.

## Methods

### Data sources

This study uses data from the following sources: Behavioral Risk Factor Surveillance System (BRFSS) data from 1985 to 2016 for alcohol outcomes, race/ethnicity, and individual-level control variables; NIAAA's Alcohol Policy Information System (APIS) and other legal databases for alcohol/pregnancy policies; and secondary sources for state-level characteristics.

#### Behavioral risk factor surveillance system

BRFSS is an annual telephone survey that tracks health status and health behaviors of U.S. adults. It has been conducted annually since 1984, with pregnancy status assessed since 1985. BRFSS has included alcohol use questions since the first survey, although alcohol data were not collected during even years in the 1990s. The CDC has used BRFSS data for national estimates of drinking during pregnancy since 1991.^5–7^ Participation rates were more than 70% in 1993 and closer to 50% through the 2000s. Our analytic sample consists of female BRFSS respondents of reproductive age (age 18–44) who indicated that they were currently pregnant and provided data on drinking, pooled across 1985–2016 (*N*=57,194).

#### APIS and legal databases

APIS provides information on alcohol-related policies in the United States.^24^ Specific alcohol/pregnancy policies tracked in APIS include policies pertaining to CC, legal significance for CACN, PCP, PTP, PTPWC, RRDTx, RRCPS, and MWS. APIS data were augmented with original legal research using Westlaw and HeinOnline, two online legal databases. The process for obtaining and coding these data is detailed elsewhere.^11^

#### State-level characteristics

State-level characteristics were obtained from secondary sources, including U.S. census, U.S. CDC, APIS, National Highway Traffic Safety Administration, National Beverage Control Association, published research,^25^ and original legal research.

### Measures

#### Drinking

Drinking outcomes were selected based on the official U.S. recommendation of abstinence from alcohol use during pregnancy and literature finding increased risks of poor outcomes with binge and higher volume drinking.^2,3^ BRFSS alcohol consumption data are measured for past 30 days. We created indicators of: (1) any drinking (dichotomous, one or more drinks); (2) binge drinking [dichotomous, five or more (four or more beginning in 2006) drinks on an occasion]; and (3) heavy drinking [based on frequency, quantity, and binge frequency, using indexing^26-27^ indicated by 16+ past month drinks, roughly 4 or more drinks per week, a level where there is well documented harm.^2^] Although alcohol consumption questions were asked consistently, there were some question wording changes over time. Our modeling approach (fixed effects for year) controls for measurement changes.

#### Alcohol/pregnancy policies

State-level alcohol/pregnancy policies included the five supportive and three punitive policies described earlier. Each policy is dichotomous, coded as 0 if it was not in effect for a state in a given year and 1 if it was in effect for a state in a given year.

#### Race/ethnicity

BRFSS queries both ethnicity and race; responses were used to categorize respondents as: White (reference), Black, Hispanic, or Other. The Other category included non-Hispanic respondents who endorsed Asian/Pacific Islander, Native American/Alaska Native, Mixed race, or Other and those for whom race was missing (including Don't Know).

#### Controls

Individual-level covariates from BRFSS included age (categorical, 18–24, 25–29, 30–34, 35–39, 40–45, missing), marital status (categorical, married, divorced/widowed/separated, single, member of unmarried couple), education (categorical, less than high school, high school graduate, some college, college graduate, missing), income (categorical, in 2013 $: ≤27,000, >27,000 to ≤49,000, >49,000 to ≤88,500, >88,000, missing), tobacco (categorical, no, yes, missing), and physical activity (categorical, no, yes, missing).

#### Controls

State-level covariates included state- and year-specific poverty (continuous), per capita cigarette sales (continuous, proxy for effective tobacco policies), and two general population alcohol policies on which data are available for the study time period: Blood Alcohol Concentration laws (categorical, neither a .10 nor .08 law, .10 law, .08 law) and indicators for states with retail monopolies on wine or spirits (dichotomous).

### Statistical analyses

Although our sample included over 57,000 pregnant women, small cell sizes for some racial/ethnic categories, rare drinking outcomes, and the overall complexity of models prohibited running models stratified by race/ethnicity, an approach that could help isolate effects of policies for specific subgroups. Instead, to illustrate effects for subgroups, we calculated predicted probabilities/predictive margins of outcomes when policies were and were not in effect and confidence intervals around average marginal effects (MEs) of policies by race/ethnicity in sample-weighted logistic regression models testing interactions of each policy and race/ethnicity for each outcome.

Models (24 total) included fixed effects for state and for year and adjusted for individual- and state-level covariates, including all other alcohol/pregnancy policies. Consistent with prior research examining effects of state-level policies on individual behavior with BRFSS data, standard errors were adjusted to reflect clustering by state.^28,29^ Model coefficients were expressed as odds ratios (ORs), and we conducted Wald tests (χ^2^ distribution with 3 degrees of freedom) to examine overall effects of interaction terms and to determine whether differential effects of policies by race/ethnicity were present. Analyses were conducted in Stata v.15.

## Results

The majority of the analytic sample was White (70.1%); the next largest racial/ethnicity category was Hispanic (11.9%). The remainder was split between Black (9.4%) and the Other category (8.7%). A little more than a 10th of the sample (11.1%) endorsed any drinking; 2.2% endorsed binge drinking; and 2.2% heavy drinking. About half (47.2%) were living in states when RRDTx were in effect; 6.7% were living in states when CC policies were in effect. The percent exposed to other policies was in between. The unweighted distribution of the sample by race/ethnicity and policy exposure for each outcome is shown in Supplementary Tables 1 and 2.

### Supportive policies

Table 2 displays predicted probabilities and MEs from models examining interactions between race/ethnicity and each supportive policy for each drinking outcome. ORs for interaction terms are shown in Table 3.

**Table 2.** Predicted Probability of Drinking Outcomes and Marginal Effects of Supportive Policies by Race/Ethnicity

	Any drinking				Binge drinking				Heavy drinking			
	No policy	Policy	ME	95% CI	No policy	Policy	ME	95% CI	No policy	Policy	ME	95% CI
MWS												
White	0.125	0.123	−0.002	[−0.027 to 0.022]	**0.028**	**0.017**	−**0.010**	**[**−**0.020** to −**0.001]**	0.027	0.021	−0.006	[−0.018 to 0.005]
Black	0.141	0.136	−0.005	[−0.048 to 0.038]	0.024	0.019	−0.005	[−0.019 to 0.008]	0.027	0.018	−0.010	[−0.024 to 0.005]
Hispanic	0.139	0.132	−0.007	[−0.035 to 0.021]	**0.046**	**0.021**	−**0.026**	**[**−**0.040** to −**0.011]**	0.031	0.021	−0.010	[−0.023 to 0.003]
Other	0.119	0.115	−0.004	[−0.027 to 0.018]	**0.031**	**0.016**	−**0.015**	**[**−**0.026** to −**0.004]**	0.021	0.018	−0.003	[−0.018 to 0.013]
PTP												
White	0.125	0.122	−0.003	[−0.032 to 0.026]	0.023	0.019	−0.004	[−0.015 to 0.007]	0.024	0.024	0.000	[−0.011 to 0.011]
Black	**0.132**	**0.168**	**0.036**	**[0.007** to **0.065]**	0.019	0.030	0.010	[−0.007 to 0.028]	0.022	0.027	0.005	[−0.009 to 0.019]
Hispanic	**0.149**	**0.110**	−**0.039**	**[**−**0.068** to −**0.010]**	0.036	0.021	−0.015	[−0.032 to 0.002]	0.027	0.022	−0.005	[−0.017 to 0.008]
Other	0.123	0.101	−0.022	[−0.044 to 0.000]	0.026	0.014	−0.012	[−0.026 to 0.003]	0.020	0.017	−0.003	[−0.017 to 0.011]
PTPWC												
White	**0.122**	**0.142**	**0.020**	**[0.003** to **0.037]**	0.023	0.019	−0.004	[−0.011 to 0.003]	0.025	0.017	−0.008	[−0.019 to 0.003]
Black	**0.141**	**0.111**	−**0.030**	**[**−**0.048** to −**0.012]**	0.022	0.012	−0.010	[−0.024 to 0.003]	**0.024**	**0.011**	−**0.014**	**[**−**0.025** to −**0.002]**
Hispanic	**0.131**	**0.169**	**0.038**	**[0.009** to **0.066]**	0.031	0.025	−0.006	[−0.024 to 0.011]	0.027	0.016	−0.011	[−0.028 to 0.006]
Other	0.115	0.138	0.023	[−0.002 to 0.048]	0.023	0.019	−0.004	[−0.027 to 0.018]	0.020	0.017	−0.003	[−0.019 to 0.013]
RRDTx												
White	0.127	0.121	−0.006	[−0.023 to 0.011]	0.021	0.024	0.003	[−0.003 to 0.009]	0.026	0.023	−0.002	[−0.009 to 0.004]
Black	0.135	0.143	0.007	[−0.029 to 0.044]	0.021	0.022	0.001	[−0.015 to 0.017]	0.026	0.019	−0.007	[−0.021 to 0.007]
Hispanic	0.139	0.134	−0.005	[−0.045 to 0.035]	0.037	0.028	−0.008	[−0.027 to 0.010]	0.030	0.022	−0.008	[−0.023 to 0.008]
Other	0.108	0.123	0.015	[−0.016 to 0.046]	0.030	0.019	−0.011	[−0.030 to 0.008]	0.019	0.020	0.001	[−0.017 to 0.018]
PCP												
White	**0.126**	**0.096**	−**0.030**	**[**−**0.053** to −**0.007]**	0.022	0.021	−0.001	[−0.014 to 0.011]	**0.025**	**0.015**	−**0.010**	**[**−**0.016** to −**0.002]**
Black	**0.140**	**0.107**	−**0.034**	**[**−**0.067** to −**0.001]**	0.021	0.022	0.001	[−0.013 to 0.015]	0.022	0.031	0.008	[−0.013 to 0.030]
Hispanic	0.136	0.126	−0.011	[−0.052 to 0.031]	0.031	0.024	−0.007	[−0.026 to 0.013]	0.025	0.027	0.001	[−0.019 to 0.022]
Other	0.118	0.106	−0.011	[−0.053 to 0.030]	**0.021**	**0.067**	**0.046**	**[0.002** to **0.090]**	0.018	0.049	0.030	[−0.020 to 0.081]

These models display the predicted probability (predictive margins) of outcomes based on models testing the interaction of each policy and race/ethnicity in separate sample-weighted logistic regression in models that included fixed effects for state and for year and adjusted for individual- and state-level covariates, including all other pregnancy-specific alcohol policies.

ME, (average) marginal effect.

Bold indicates statistical significance, i.e. that the ME doesnot cross 0.

**Table 3.** Interactions of Supportive Pregnancy-Specific Alcohol Policies and Race/Ethnicity by Drinking Outcome

	Any drinking			Binge drinking			Heavy drinking		
	aOR	95% CI	*p*	aOR	95% CI	*p*	aOR	95% CI	*p*
MWS	0.98	[0.76–1.25]	0.852	0.61	[0.39–0.93]	0.023	0.75	[0.44–1.25]	0.268
Race/ethnicity									
White (reference)									
Black	1.16	[0.96–1.42]	0.134	0.87	[0.60–1.26]	0.465	0.99	[0.60–1.64]	0.979
Hispanic	1.15	[0.88–1.49]	0.305	1.79	[1.14–2.79]	0.011	1.14	[0.72–1.81]	0.578
Other	0.94	[0.77–1.15]	0.564	1.14	[0.65–1.98]	0.643	0.73	[0.33–1.63]	0.446
MWS×race/ethnicity									
White (reference)									
Black	0.98	[0.72–1.33]	0.886	1.25	[0.67–2.34]	0.478	0.84	[0.44–1.60]	0.588
Hispanic	0.96	[0.73–1.26]	0.772	0.67	[0.43–1.04]	0.072	0.87	[0.54–1.39]	0.552
Other	0.98	[0.76–1.25]	0.844	0.81	[0.52–1.25]	0.332	1.13	[0.58–2.20]	0.710
Wald Test of MWS×race/ethnicity	χ^2^(3)=0.11		0.990	χ^2^(3)=5.69		0.128	χ^2^(3)=0.87		0.834
Priority Tx for pregnant women	0.97	[0.72–1.30]	0.825	0.81	[0.46–1.44]	0.472	0.99	[0.59–1.64]	0.955
Race/ethnicity									
White (reference)									
Black	1.07	[0.91–1.27]	0.392	0.82	[0.56–1.19]	0.287	0.88	[0.51–1.51]	0.640
Hispanic	1.26	[1.07–1.49]	0.007	1.61	[1.02–2.55]	0.043	1.12	[0.74–1.71]	0.589
Other	0.98	[0.82–1.17]	0.818	1.14	[0.70–1.85]	0.594	0.82	[0.43–1.56]	0.541
PTP×race/ethnicity									
White (reference)									
Black	1.42	[1.02–1.98]	0.038	1.98	[0.82–4.80]	0.129	1.29	[0.71–2.33]	0.405
Hispanic	0.70	[0.57–0.86]	0.001	0.68	[0.42–1.12]	0.134	0.82	[0.51–1.30]	0.399
Other	0.81	[0.65–1.00]	0.055	0.65	[0.35–1.21]	0.178	0.86	[0.44–1.66]	0.647
Wald Test of PTP×race/ethnicity	**χ^2^(3)=25.36**		**0.000**	**χ^2^(3)=6.77**		**0.080**	χ^2^(3)=3.13		0.372
PTPWC×race/ethnicity	1.22	[1.04–1.43]	0.015	0.81	[0.54–1.21]	0.308	0.66	[0.33–1.32]	0.244
Race/ethnicity									
White (reference)									
Black	1.21	[1.04–1.40]	0.013	0.99	[0.71–1.37]	0.961	0.96	[0.60–1.52]	0.853
Hispanic	1.10	[0.91–1.32]	0.318	1.44	[0.96–2.15]	0.081	1.07	[0.80–1.43]	0.659
Other	0.93	[0.78–1.10]	0.387	1.04	[0.58–1.85]	0.905	0.77	[0.46–1.28]	0.315
PTPWC×race/ethnicity									
White (reference)									
Black	0.61	[0.51–0.72]	0.000	0.63	[0.23–1.60]	0.362	0.62	[0.23–1.67]	0.348
Hispanic	1.14	[0.87–1.51]	0.342	0.97	[0.50–1.89]	0.921	0.85	[0.19–3.73]	0.827
Other	1.04	[0.81–1.32]	0.775	0.98	[0.29–3.32]	0.979	1.27	[0.48–3.32]	0.630
Wald Test of PTPWC×race/ethnicity	**χ^2^(3)=37.75**		**0.000**	χ^2^(3)=1.90		0.594	χ^2^(3)=2.87		0.412
RRDTx	0.94	[0.79–1.12]	0.506	1.14	[0.84–1.54]	0.395	0.89	[0.68–1.18]	0.428
Race/ethnicity									
White (reference)									
Black	1.09	[0.90–1.32]	0.390	1.00	[0.57–1.75]	0.998	1.02	[0.57–1.83]	0.952
Hispanic	1.12	[0.83–1.51]	0.446	1.88	[1.02–3.46]	0.043	1.19	[0.67–2.11]	0.545
Other	0.82	[0.61–1.10]	0.193	1.47	[0.66–3.28]	0.350	0.72	[0.25–2.04]	0.531
RRDTx×race/ethnicity									
White (reference)									
Black	1.14	[0.80–1.61]	0.470	0.93	[0.37–2.32]	0.879	0.79	[0.38–1.63]	0.518
Hispanic	1.01	[0.75–1.37]	0.932	0.66	[0.36–1.19]	0.167	0.80	[0.44–1.47]	0.481
Other	1.25	[0.92–1.70]	0.156	0.53	[0.27–1.04]	0.063	1.18	[0.40–3.48]	0.769
Wald Test of RRDTx×race/ethnicity	χ^2^(3)=2.02		0.568	**χ^2^(3)=6.74**		**0.081**	χ^2^(3)=1.12		0.771
PCP	0.72	[0.54–0.95]	0.022	0.93	[0.48–1.80]	0.824	0.59	[0.37–0.95]	0.031
Race/ethnicity									
White (reference)									
Black	1.15	[0.98–1.36]	0.081	0.96	[0.69–1.33]	0.800	0.90	[0.56–1.43]	0.645
Hispanic	1.11	[0.93–1.32]	0.230	1.44	[0.96–2.15]	0.076	1.03	[0.73–1.44]	0.881
Other	0.92	[0.78–1.08]	0.331	0.96	[0.56–1.65]	0.886	0.72	[0.44–1.20]	0.215
PCP×race/ethnicity									
White (reference)									
Black	0.99	[0.74–1.31]	0.947	1.14	[0.53–2.43]	0.739	2.38	[0.89–6.41]	0.085
Hispanic	1.26	[0.87–1.83]	0.218	0.82	[0.35–1.96]	0.663	1.80	[0.71–4.57]	0.218
Other	1.23	[0.78–1.97]	0.365	3.98	[1.84–8.63]	0.000	4.97	[1.65–14.93]	0.004
Wald Test of PCP×race/ethnicity	χ^2^(3)=2.54		0.468	**χ^2^(3)=16.54**		**0.001**	**χ^2^(3)=17.37**		**0.001**

Individuals in the Other category are those who endorsed Asian/Pacific Islander, Native American/Alaskan Native, Mixed, and Other, as well as those who did not answer this question. Models tested the full factorial interaction of race/ethnicity and each of the supportive policies separately. All models adjusted for state and time fixed effects, as well as individual and state-level covariates (including all other pregnancy-specific alcohol policies). Models also include BRFSS sample weights and adjusted standard errors to reflect clustering at the state level. The Wald test uses the Chi-square distribution with 3 degrees of freedom and tests the hypothesis that all coefficients in the interaction term are 0.

aoR, adjusted odds Ratio; BRFSS, Behavioral Risk Factor Surveillance System.

#### Mandatory warning signs

Table 2 shows that MWS were associated with significant reductions in probabilities of binge drinking among Whites (ME=−0.010, *p*=0.037), Hispanics (ME=−0.026, *p*=0.001), and the Other category (ME=−0.015, *p*=0.010). As indicated by the main effect of MWS in Table 3, MWS were associated with decreased odds of binge drinking among Whites adjusted odds ratio (aOR=0.61, *p*=0.023). Although the ME on binge drinking was greatest among Hispanics, the relationship between MWS and binge drinking was only marginally significant relative to Whites (aOR=0.67, *p*=0.072), and the overall effect of the interaction was not statistically significant.

#### Priority treatment for pregnant women

PTP was associated with increased probability of any drinking among Blacks (ME=0.036, *p*=0.014) and decreased probability of any drinking among Hispanics (ME=−0.039, *p*=0.008). Based on the overall test of the interaction, there was evidence of differential effects of PTP on any drinking [χ^2^(3)=25.36, *p*<0.001]. Relative to Whites, PTP was associated with increased odds of any drinking among Blacks (aOR=1.42, *p*=0.038) and decreased odds of any drinking among Hispanics (aOR=0.70, *p*=0.001).

#### Priority treatment for pregnant women and women with children

PTPWC was associated with increased probabilities of any drinking among Whites (ME=0.020, *p*=0.018) and Hispanics (ME=0.038, *p*=0.009) but decreased probability of any drinking among Blacks (ME=−0.030, *p*=0.001). PTPWC was also associated with decreased probability of heavy drinking among Blacks (ME=−0.014, *p*=0.017). There was evidence of differential effects by race/ethnicity on any drinking [χ^2^(3)=37.75, *p*<0.001]. As indicated by a significant main effect, PTPWC was associated with increased odds of any drinking for Whites (aOR=1.22, *p*=0.015). Relative to Whites, PTPWC was associated with decreased odds of any drinking among Blacks (aOR=0.60, *p*<0.001). Although the increased probability of any drinking was largest among Hispanics, PTPWC was not associated with significantly increased odds of any drinking among Hispanics relative to Whites. There was no evidence of differential effects of PTPWC by race/ethnicity on heavy drinking, despite the significant ME for Blacks.

#### Prohibitions on criminal prosecution

PCP were associated with significantly decreased probability of any and heavy drinking among Whites (ME=−0.030, *p*=0.012; ME=−0.010, *p*=0.008, respectively). They were also associated with decreased probability of any drinking among Blacks (ME=−0.034, *p*=0.045) and increased probability of binge drinking among the Other category (ME=0.046, *p*=0.042). As indicated by a significant main effect, PCP were associated with decreased odds of any drinking among Whites (aOR=0.72, *p*=0.022), but there was no evidence to suggest differential effects on any drinking by race/ethnicity based on the test of the interaction.

There were significant interactions between PCP and race/ethnicity for both binge [χ^2^(3)=16.54, *p*=0.001] and heavy drinking [χ^2^(3)=17.37, *p*=0.001]. Relative to Whites, PCP among the Other category were associated with increased odds of binge drinking (aOR=3.98, *p*<0.001). As indicated by a significant main effect, PCP were associated with decreased odds of heavy drinking among Whites (aOR=0.59, *p*=0.031); relative to Whites, PCP were associated with increased odds of heavy drinking among those in the Other category (aOR=4.97, *p*=0.004).

### Punitive policies

Table 4 displays predicted probabilities and MEs from models examining interactions between race/ethnicity and each punitive policy for each drinking outcome. ORs for interaction terms are shown in Table 5.

**Table 4.** Predicted Probability of Drinking Outcomes and Marginal Effects of Punitive Policies by Race/Ethnicity

	Any drinking				Binge drinking				Heavy drinking			
	No policy	Policy	ME	95% CI	No policy	Policy	ME	95% CI	No policy	Policy	ME	95% CI
CACN												
White	0.122	0.130	0.008	[−0.015 to 0.030]	0.024	0.018	−0.005	[−0.013 to 0.002]	0.026	0.020	−0.006	[−0.015 to 0.003]
Black	0.145	0.118	−0.027	[−0.066 to 0.012]	**0.026**	**0.012**	−**0.013**	**[**−**0.023** to −**0.004]**	**0.027**	**0.013**	−**0.014**	**[**−**0.026** to −**0.002]**
Hispanic	0.133	0.140	0.007	[−0.023 to 0.037]	0.035	0.023	−0.012	[−0.026 to 0.002]	**0.030**	**0.015**	−**0.015**	**[**−**0.025** to −**0.004]**
Other	0.115	0.123	0.008	[−0.026 to 0.042]	0.023	0.023	0.000	[−0.016 to 0.016]	0.022	0.014	−0.008	[−0.023 to 0.007]
CC												
White	0.124	0.105	−0.019	[−0.050 to 0.012]	0.022	0.035	0.013	[−0.006 to 0.032]	0.024	0.041	0.017	[−0.013 to 0.047]
Black	0.139	0.118	−0.021	[−0.079 to 0.037]	0.021	0.051	0.030	[−0.025 to 0.086]	0.022	0.079	0.057	[−0.024 to 0.138]
Hispanic	**0.137**	**0.070**	−**0.067**	**[**−**0.133** to −**0.001]**	**0.031**	**0.007**	−**0.024**	**[**−**0.033** to −**0.016]**	0.026	0.015	−0.010	[−0.029 to 0.008]
Other	0.118	0.079	−0.039	[−0.062 to 0.042]	0.023	0.029	0.006	[−0.016 to 0.024]	0.020	0.025	0.005	[−0.023 to 0.035]
Reporting requirements for CPS												
White	0.125	0.121	−0.005	[−0.022 to 0.013]	0.021	0.025	0.004	[−0.004 to 0.013]	0.024	0.025	0.001	[−0.008 to 0.011]
Black	0.138	0.145	0.007	[−0.034 to 0.049]	0.018	0.031	0.013	[−0.007 to 0.032]	0.022	0.024	0.002	[−0.018 to 0.021]
Hispanic	0.142	0.125	−0.017	[−0.043 to 0.010]	0.029	0.035	0.006	[−0.009 to 0.021]	0.024	0.029	0.005	[−0.009 to 0.018]
Other	0.118	0.114	−0.004	[−0.030 to 0.022]	0.023	0.023	0.000	[−0.012 to 0.012]	0.019	0.022	0.003	[−0.010 to 0.017]

These models display the predicted probability (predicted margins) of outcomes based on models testing the interaction of each policy and race/ethnicity in separate sample-weighted logistic regression in models that included fixed effects for state and for year and adjusted for individual- and state-level covariates, including all other pregnancy-specific alcohol policies.

**Table 5.** Interactions of Punitive Pregnancy-Specific Alcohol Policies and Race/Ethnicity by Drinking Outcome

	Any drinking			Binge drinking			Heavy drinking		
	OR	95% CI	*p*	OR	95% CI	*p*	OR	95% CI	*p*
CACN	1.08	[0.86–1.35]	0.490	0.75	[0.51–1.11]	0.157	0.75	[0.48–1.15]	0.186
Race/ethnicity									
White (reference)									
Black	1.24	[1.07–1.45]	0.005	1.07	[0.74–1.56]	0.709	1.02	[0.59–1.75]	0.952
Hispanic	1.12	[0.89–1.42]	0.341	1.50	[0.90–2.51]	0.119	1.18	[0.82–1.68]	0.369
Other	0.93	[0.76–1.14]	0.476	0.95	[0.47–1.93]	0.890	0.82	[0.44–1.53]	0.535
CACN×race/ethnicity									
White (reference)									
Black	0.71	[0.51–0.99]	0.043	0.60	[0.30–1.20]	0.151	0.61	[0.27–1.37]	0.229
Hispanic	0.99	[0.74–1.31]	0.928	0.84	[0.42–1.68]	0.622	0.65	[0.33–1.25]	0.192
Other	1.01	[0.74–1.38]	0.963	1.34	[0.55–3.24]	0.515	0.83	[0.35–1.94]	0.659
Wald Test of CACN×race/ethnicity	**χ^2^(3)=6.66**		**0.084**	χ^2^(3)=3.78		0.286	χ^2^(3)=2.16		0.541
CC	0.81	[0.56–1.17]	0.259	1.68	[0.90–3.16]	0.104	1.82	[0.77–4.28]	0.171
Race/ethnicity									
White (reference)									
Black	1.16	[0.99–1.34]	0.065	0.95	[0.69–1.31]	0.770	0.91	[0.57–1.45]	0.693
Hispanic	1.13	[0.95–1.35]	0.164	1.48	[1.00–2.20]	0.053	1.07	[0.76–1.49]	0.706
Other	0.94	[0.79–1.11]	0.459	1.05	[0.60–1.83]	0.865	0.80	[0.47–1.33]	0.386
CC×race/ethnicity									
White (reference)									
Black	1.00	[0.42–2.38]	0.994	1.63	[0.43–6.15]	0.474	2.42	[0.65–8.93]	0.186
Hispanic	0.54	[0.14–2.05]	0.369	0.12	[0.08–0.17]	0.000	0.31	[0.20–0.50]	0.000
Other	0.75	[0.52–1.08]	0.127	0.76	[0.34–1.70]	0.507	0.72	[0.22–2.30]	0.578
Wald Test of CC×race/ethnicity	χ^2^(3)=4.02		0.259	**χ^2^(3)=146.49**		**0.000**	**χ^2^(3)=24.30**		**0.000**
CPS RR	0.95	[0.80–1.14]	0.614	1.22	[0.82–1.81]	0.325	1.06	[0.70–1.60]	0.777
Race/ethnicity									
White (reference)									
Black	1.13	[0.95–1.34]	0.172	0.87	[0.56–1.34]	0.524	0.92	[0.52–1.63]	0.779
Hispanic	1.18	[1.00–1.38]	0.054	1.43	[0.87–2.35]	0.160	1.00	[0.62–1.59]	0.994
Other	0.93	[0.75–1.15]	0.499	1.11	[0.64–1.93]	0.711	0.76	[0.36–1.58]	0.458
CPS RR×race/ethnicity									
White (reference)									
Black	1.12	[0.82–1.54]	0.482	1.45	[0.58–3.63]	0.422	1.03	[0.44–2.40]	0.944
Hispanic	0.89	[0.72–1.10]	0.283	1.01	[0.61–1.69]	0.961	1.15	[0.73–1.82]	0.547
Other	1.00	[0.81–1.24]	0.970	0.82	[0.46–1.47]	0.501	1.13	[0.54–2.40]	0.743
Wald Test of CPS RR×race/ethnicity	χ^2^(3)=1.56		0.668	χ^2^(3)=1.07		0.783	χ^2^(3)=0.75		0.861

Individuals in the Other category are those who endorsed Asian/Pacific Islander, Native American/Alaskan Native, Mixed, and Other, as well as those who did not answer this question. Models tested the interaction of race/ethnicity on all eight policies separately and adjusted for state and time fixed effects, as well as individual and state-level covariates (including all other pregnancy-specific alcohol policies). Models also include BRFSS sample weights and adjusted standard errors to reflect clustering at the state level. The Wald test uses the Chi-square distribution with 3 degrees of freedom and tests the hypothesis that all coefficients in the interaction term are 0.

OR, odds ratio.

#### Child abuse/neglect

CACN were associated with significantly decreased probabilities of binge and heavy drinking among Blacks (ME=−0.013, *p*=0.005; ME=−0.014, *p*=0.022, respectively) and decreased heavy drinking among Hispanics (ME=−0.015, *p*=0.005). There was some evidence to suggest potential differential effects. Relative to Whites, CACN were associated with decreased odds of any drinking among Blacks (aOR=0.71, *p*=0.043), but the Wald test of the interaction was marginally significant [χ^2^(3)=6.66, *p*=0.084].

#### Civil commitment

CC was associated with significantly decreased probabilities of any and binge drinking among Hispanics (ME=−0.067, *p*=0.047; ME=−0.024, *p*<0.001, respectively) Significant interactions were present in models examining binge [χ^2^(3)=146.49, *p*<0.001] and heavy drinking [χ^2^(3)=24.30, *p*<0.001]. Relative to Whites, CC was associated with decreased odds of binge drinking (aOR=0.12, *p*<0.001) among Hispanics. CC was also associated with a decreased odds of heavy drinking among Hispanics (aOR=0.31, *p*<0.001) relative to Whites.

## Discussion

This study examined differential effects of alcohol/pregnancy policies by race/ethnicity and contributes two main findings. First, differential effects by race/ethnicity appear to mask overall effects. Second, health benefits/harms from alcohol/pregnancy policies are not equally distributed across White, Black, and Hispanic women.

Regarding differential effects masking overall effects, we find examples of no effect in previous analyses due to effects in opposite directions and effects concentrated in subgroups. While there was no effect for PTP overall, effects in opposite directions appear to mask overall effects. The lack of effects for PCP and CC overall appears due, in part, to effects concentrated among subgroups. These findings confirm the importance of subgroup analyses in policy impact research, especially related to alcohol during pregnancy.

We expected health benefits from alcohol/pregnancy policies (i.e., reduced drinking during pregnancy) to be stronger for White than Black and Hispanic women and health harms from these policies (i.e., increased drinking during pregnancy) to be stronger for Black and Hispanic than White women. Some findings for supportive policies support this pattern, for example, White, but not Black, women reporting less binge drinking when MWS were in effect (although interaction term is not significant); Black, but not White, women reporting more any drinking when PTP is in effect; and White and Black, but not Hispanic, women reporting less any drinking when PCP is in effect (although only White women report less heavy drinking when PCP is in effect). Other findings do not support this pattern: Hispanic women benefited similarly to White women when MWS were in effect and benefited more than White women when PTP was in effect. This pattern suggests that the general hypothesis may apply for White versus Black women for supportive policies, but does not apply to Hispanic women.

For punitive policies, however, findings do not support this hypothesis. While White women's drinking is not affected by CACN or CC, Black and Hispanic women reported less drinking when CACN policies were in effect and Hispanic women reported less drinking when CC policies were in effect. This pattern suggests that Black and Hispanic women, who may be more likely than White women to be punished for alcohol use during pregnancy,^21–23^ respond to this threat by reducing drinking. Black and Hispanic women could also be less likely to report their drinking when these policies are in effect, out of fear of punishment. This explanation is supported by previous research that found increased adverse birth outcomes and decreased prenatal care utilization when punitive policies were in effect.^30^ Future research using measures beyond self-reports can help disentangle this pattern. These findings must be interpreted with caution due to smaller proportion of Hispanics in the sample and the relative rarity of CC policies. Furthermore, CACN and CC policies are problematic on ethical grounds^31^; any reductions in self-reported drinking with respect to punitive policies must be investigated with more robust measures of health outcomes and other measures of health benefits that may offset inherent harms.

While not related to main hypotheses, the pattern of findings for different Priority Treatment policies is worth noting. In addition to findings in opposite directions across race/ethnicity for PTP only, there were also findings in opposite directions across race/ethnicity for PTPWC. However, patterns differed across priority treatment policies; White and Black women benefited from different priority treatment policies. This suggests that different priority treatment policies benefit versus harm different subgroups, perhaps by giving priority to some groups but not others, and that bringing children to treatment may be especially important for Black women. Additional research could explain this pattern and assess whether the pattern could be addressed through other mechanisms, such as increasing treatment availability for pregnant women.

To our knowledge, this is the first study of differential effects of a range of alcohol/pregnancy policies on drinking behavior by race/ethnicity, despite such policies being in effect for over four decades. Limitations include limited numbers of pregnant women in some racial/ethnic categories exposed to some policies; self-reported alcohol use measures that could involve differential underreporting of alcohol use due to social characteristics and studied policies; and no measures of policy enforcement or women's awareness of policies. In addition, alcohol use during pregnancy is not the only health outcome related to alcohol/pregnancy policies. Research should examine differential effects on outcomes such as prenatal care, treatment utilization, and birth outcomes.

## Conclusions

Research considering the impact of alcohol/pregnancy policies should consider differential effects by race/ethnicity, as subgroup analyses indicate that benefits and harms of policies do not appear equitably distributed across race/ethnicity.

## Supplementary Material

Supplemental data

Supplemental data

## Abbreviations Used

aOR: adjusted odds ratio
APIS: Alcohol Policy Information System
BRFSS: Behavioral Risk Factor Surveillance System
CACN: child abuse/neglect
CC: civil commitment
CPS: child protective services
MEs: marginal effects
MWS: mandatory warning signs
OR: odds ratio
PCP: prohibitions on criminal prosecution
PTP: priority treatment for pregnant women
PTPWC: priority treatment for pregnant women and women with children
RRDTx: reporting requirements for data and treatment purposes
RRCPS: reporting requirements for child protective services purposes

## References

[1] Sokol RJ, Delaney-Black V, Nordstrom B Fetal alcohol spectrum disorder. JAMA. 2003;290:2996–2999 14665662 10.1001/jama.290.22.2996

[2] O'Leary CM, Bower C Guidelines for pregnancy: what's an acceptable risk, and how is the evidence (finally) shaping up? Drug Alcohol Rev. 2012;31:170–183 21955332 10.1111/j.1465-3362.2011.00331.x

[3] Sayal K, Heron J, Golding J, et al. Binge pattern of alcohol consumption during pregnancy and childhood mental health outcomes: longitudinal population-based study. Pediatrics. 2009;123:e289–e296 19171582 10.1542/peds.2008-1861 PMC6485410

[4] Popova S, Lange S, Probst C, et al. Estimation of national, regional, and global prevalence of alcohol use during pregnancy and fetal alcohol syndrome: a systematic review and meta-analysis. Lancet Glob Health. 2017;5:e290–e299 28089487 10.1016/S2214-109X(17)30021-9

[5] Centers for Disease Control and Prevention. Alcohol consumption among pregnant and childbearing-aged women—United States, 1991 and 1995. MMWR Morb Mortal Wkly Rep. 1997;46:346–350 9148136

[6] Centers for Disease C, Prevention. Alcohol use among women of childbearing age—United States, 1991–1999. MMWR Morb Mortal Wkly Rep. 2002;51:273–276 11952279

[7] Centers for Disease Control and Prevention. Alcohol use among pregnant and nonpregnant women of childbearing age—United States, 1991–2005. MMWR Morb Mortal Wkly Rep. 2009;58:529–532 19478721

[8] Serdula M, Williamson DF, Kendrick JS, et al. Trends in alcohol consumption by pregnant women. 1985 through 1988. JAMA. 1991;265:876–879 1992184

[9] Zhao G, Ford ES, Tsai J, et al. Trends in health-related behavioral risk factors among pregnant women in the United States: 2001–2009. J Women's Health. 2012;21:255–263 10.1089/jwh.2011.2931 22047097

[10] Suellentrop K, Morrow B, Williams L, et al. Monitoring progress toward achieving maternal and infant healthy people 2010 objectives—19 states, Pregnancy Risk Assessment Monitoring System (PRAMS), 2000–2003. MMWR Morb Mortal Wkly Rep. 2006;55:1–11 17021594

[11] Roberts SCM, Thomas S, Treffers R, et al. Forty years of state alcohol and pregnancy policies in the USA: best practices for public health or efforts to restrict women's reproductive rights? Alcohol Alcohol. 2017;52:715–721 29016712 10.1093/alcalc/agx047 PMC5860145

[12] Gomez LE Misconceiving Mothers: Legislators, Prosecutors, and the Politics of Prenatal Drug Exposure. Philadelphia: Temple University Press, 1997

[13] Chavkin W, Wise PH, Elman D Policies towards pregnancy and addiction. Sticks without carrots. Ann N Y Acad Sci. 1998;846:335–340 9668420

[14] Roberts SCM, Pies C Complex calculations: how drug use during pregnancy becomes a barrier to prenatal care. Matern Child Health J. 2011;15:333–341 20232126 10.1007/s10995-010-0594-7 PMC2904854

[15] Stone R Pregnant women and substance use: fear, stigma, and barriers to care. Health Justice. 2015;3:2

[16] Cil G Effects of posted point-of-sale warnings on alcohol consumption during pregnancy and on birth outcomes. J Health Econ. 2017;53:131–155 28343094 10.1016/j.jhealeco.2017.03.004

[17] Keppel K, Bilheimer L, Gurley L Improving population health and reducing health care disparities. Health Aff. 2007;26:1281–1292 10.1377/hlthaff.26.5.1281 17848438

[18] Sumar N, McLaren L Impact on social inequalities of population strategies of prevention for folate intake in women of childbearing age. Am J Public Health. 2011;101:1218–1224 21566037 10.2105/AJPH.2010.300018 PMC3110217

[19] McLaren L, McIntyre L, Kirkpatrick S Rose's population strategy of prevention need not increase social inequalities in health. Int J Epidemiol. 2010;39:372–377 19887510 10.1093/ije/dyp315

[20] Frohlich KL, Potvin L Transcending the known in public health practice: the inequality paradox: the population approach and vulnerable populations. Am J Public Health. 2008;98:216–221 18172133 10.2105/AJPH.2007.114777 PMC2376882

[21] Cort NA, Cerulli C, He H Investigating health disparities and disproportionality in child maltreatment reporting: 2002–2006. J Public Health Manag Pract. 2010;16:329–336 20520372 10.1097/PHH.0b013e3181c4d933 PMC2914097

[22] Roberts SC, Nuru-Jeter A Universal screening for alcohol and drug use and racial disparities in child protective services reporting. J Behav Health Serv Res. 2012;39:3–16 21681593 10.1007/s11414-011-9247-x PMC3297420

[23] Neuspiel DR, Zingman TM, Templeton VH, et al. Custody of cocaine-exposed newborns: determinants of discharge decisions. Am J Public Health. 1993;83:1726–1729 8259803 10.2105/ajph.83.12.1726 PMC1694945

[24] NIAAA. Alcohol Policy Information System. 2016 Available at www.alcoholpolicy.niaaa.nih.gov Accessed 127, 2017

[25] Kerr WC, Williams E, Greenfield TK Analysis of price changes in Washington following the 2012 liquor privatization. Alcohol Alcohol. 2015;50:654–660 26109262 10.1093/alcalc/agv067 PMC4608622

[26] Armor DJ, Polich JM Measurement of alcohol consumption. In: Encyclopedic Handbook of Alcoholism. Edited by EM Pattison, E Kaufman New York: Gardner Press, 1982, pp. 72–80

[27] Stahre M, Naimi T, Brewer R, et al. Measuring average alcohol consumption: the impact of including binge drinks in quantity-frequency calculations. Addiction. 2006;101:1711–1718 17156170 10.1111/j.1360-0443.2006.01615.x

[28] Friedman AS, Schpero WL, Busch SH Evidence suggests that the ACA's tobacco surcharges reduced insurance take-up and did not increase smoking cessation. Health Aff. 2016;35:1176–1183 10.1377/hlthaff.2015.1540 PMC5589079 27385231

[29] McGeary KA The impact of state-level nutrition-education program funding on BMI: evidence from the behavioral risk factor surveillance system. Soc Sci Med. 2013;82:67–78 23453319 10.1016/j.socscimed.2013.01.023

[30] Subbaraman MS, Thomas S, Treffers R, et al. Associations between state-level policies regarding alcohol use among pregnant women, adverse birth outcomes, and prenatal care utilization:Results from 1972–2013 Vital Statistics. Alcohol Clin Exp Res. 2018;42:1511–1517 10.1111/acer.13804 PMC6298847 29912478

[31] Lindner EN Punishing prenatal alcohol abuse: the problems inherent in utilizing civil commitment to address addiction. Univ Illinois Law Rev. 2005;3:873–901

