# Supplementary material for: Differential Effects of Pregnancy-Specific Alcohol Policies on Drinking Among Pregnant Women by Race/Ethnicity
Source: Health Equity. 2018 Dec 13;2(1):356–65. doi: 10.1089/heq.2018.0059 (PMC6296158; doi:10.1089/heq.2018.0059)
Supplement: Supplemental data [file Supp_Table1.pdf]

Supplementary Data

Supplementary Table 1. Unweighted Distribution of Behavioral Risk Factor Surveillance System Respondents ($N = 57{,}194$) by Outcomes, Race/Ethnicity, and Supportive Policy Exposure

	Mandatory warning signs				Priority Tx for pregnant women				Priority Tx for pregnant women+				Reporting requirement for data and Tx purposes				Prohibitions on criminal prosecution			
	No policy		Policy		No policy		Policy		No policy		Policy		No policy		Policy		No policy		Policy	
**Drinking outcome**	**No**	**Yes**	**No**	**Yes**	**No**	**Yes**	**No**	**Yes**	**No**	**Yes**	**No**	**Yes**	**No**	**Yes**	**No**	**Yes**	**No**	**Yes**	**No**	**Yes**
Any drinking																				
White	21,563	2876	14,039	1596	28,874	3749	6728	723	32,142	4042	3460	430	19,090	2525	16,512	1947	32,005	4153	3597	319
Black	3021	420	1685	241	3630	491	1076	170	4169	594	537	67	2841	397	1865	264	4277	622	429	39
Hispanic	2663	321	3431	362	4349	524	1745	159	5345	611	749	72	2501	306	3593	377	5630	637	464	46
Other	2397	290	2043	236	3494	443	946	83	4012	463	428	63	2257	280	2183	246	4130	496	310	30
Binge drinking																				
White	23,911	505	15,330	297	31,936	661	7305	141	35,423	735	3818	67	21,207	397	18,034	405	35,392	740	3849	62
Black	3355	78	1865	54	4012	99	1208	33	4635	116	585	16	3156	76	2064	56	4758	126	462	6
Hispanic	2883	94	3693	98	4714	151	1862	41	5770	177	806	15	2714	88	3862	104	6075	183	501	9
Other	2606	76	2210	64	3811	119	1005	21	4337	129	479	11	2456	78	2360	62	4490	127	326	13
Heavy drinking																				
White	23,792	544	15,259	317	31,767	726	7284	135	35,249	789	3802	72	21,045	489	18,006	372	35,199	811	3852	50
Black	3335	77	1852	55	3995	94	1192	38	4605	116	582	16	3134	80	2053	52	4734	120	453	12
Hispanic	2899	63	3690	82	4723	113	1866	32	5784	132	805	13	2713	75	3876	70	6090	134	499	11
Other	2604	65	2214	48	3810	100	1008	13	4339	102	479	11	2451	65	2367	48	4488	105	330	8

This table displays how many in the analytic sample were exposed to a specific supportive policy by race for each drinking outcome. For example, among pregnant women living in states when Mandatory Warning Sign policies were in effect who reported any drinking, 1596 were White.

Tx, treatment.
