# Supplementary material for: Differential Effects of Pregnancy-Specific Alcohol Policies on Drinking Among Pregnant Women by Race/Ethnicity
Source: Health Equity. 2018 Dec 13;2(1):356–65. doi: 10.1089/heq.2018.0059 (PMC6296158; doi:10.1089/heq.2018.0059)
Supplement: Supplemental data [file Supp_Table2.pdf]

**Supplementary Table 2. Unweighted Distribution of Behavioral Risk Factor Surveillance System Respondents (*N* = 57,194) by Outcomes, Race/Ethnicity, and Punitive Policy Exposure**

| | Child abuse/neglect | | | | Civil commitment | | | | Reporting requirements for CPS | | | |
|---|---|---|---|---|---|---|---|---|---|---|---|---|
| | No policy | | Policy | | No policy | | Policy | | No policy | | Policy | |
| Drinking outcome | No | Yes | No | Yes | No | Yes | No | Yes | No | Yes | No | Yes |
| Any drinking | | | | | | | | | | | | |
| White | 23,966 | 3,248 | 11,636 | 1224 | 32,981 | 4190 | 2621 | 282 | 24,210 | 3179 | 11,392 | 1293 |
| Black | 3219 | 510 | 1487 | 151 | 4515 | 639 | 191 | 22 | 3500 | 506 | 1206 | 155 |
| Hispanic | 3616 | 442 | 2478 | 241 | 5855 | 666 | 239 | 17 | 3988 | 454 | 2106 | 229 |
| Other | 3161 | 394 | 1279 | 132 | 3997 | 492 | 443 | 34 | 3053 | 369 | 1387 | 157 |
| Binge drinking | | | | | | | | | | | | |
| White | 26,642 | 546 | 12,599 | 256 | 36,400 | 739 | 2841 | 63 | 26,857 | 510 | 12,384 | 292 |
| Black | 3613 | 104 | 1607 | 28 | 5016 | 124 | 204 | 8 | 3902 | 92 | 1318 | 40 |
| Hispanic | 3930 | 122 | 2646 | 70 | 6323 | 190 | 253 | 2 | 4314 | 121 | 2262 | 71 |
| Other | 3447 | 101 | 1369 | 39 | 4354 | 127 | 462 | 13 | 3322 | 96 | 1494 | 44 |
| Heavy drinking | | | | | | | | | | | | |
| White | 26,475 | 618 | 12,576 | 243 | 36,206 | 811 | 2845 | 50 | 26,682 | 589 | 12,369 | 272 |
| Black | 3585 | 104 | 1602 | 28 | 4982 | 125 | 205 | 7 | 3871 | 99 | 1316 | 33 |
| Hispanic | 3928 | 102 | 2661 | 43 | 6336 | 143 | 253 | 2 | 4317 | 98 | 2272 | 47 |
| Other | 3444 | 86 | 1374 | 27 | 4355 | 105 | 463 | 8 | 3322 | 80 | 1496 | 33 |

This table displays how many in the analytic sample were exposed to a specific punitive policy by race for each drinking outcome. For example, among pregnant women living in states when Civil Commitment policies were in effect who reported any drinking, 282 were White.

CPS, child protective services.
